# Concomitant upper extremity fractures do not worsen outcomes in conservatively managed pelvic fractures: a cohort study of 1840 geriatric patients

**DOI:** 10.1007/s00402-026-06444-8

**Published:** 2026-07-30

**Authors:** Nadav Graif, Etay Elbaz, Efi Kazum, Gil Rachevsky, Ron Gurel, Shai Factor

**Affiliations:** 1 Gray Faculty of Medical & Health Sciences, Tel Aviv University, Tel Aviv, Israel; 2 Orthopedic Division, Tel Aviv Sourasky Medical Center, Tel Aviv, Israel

**Keywords:** Pelvic fractures, Upper extremity fractures, Geriatric trauma, Conservative management, Mortality

## Abstract

**Introduction:**

Pelvic fractures in elderly patients are associated with significant pain, functional decline, and prolonged rehabilitation, with the majority managed non-operatively. In the conservative management paradigm, intact upper extremity function is critical for mobilization with assistive devices; concurrent upper extremity (UE) fractures may therefore complicate recovery. The aim of this study was to evaluate the clinical outcomes associated with this injury combination in patients with conservatively managed pelvic fractures.

**Methods:**

A retrospective cohort study was conducted at a Level 1 trauma center, including all patients aged ≥ 65 years admitted with pelvic ring or acetabular fractures managed non-operatively between January 2010 and January 2024. Patients were stratified by the presence of a concomitant UE fracture sustained during the same traumatic event. Primary outcomes were hospital length of stay (LOS) and all-cause mortality at multiple time points. Secondary outcomes included readmission rates and systemic infection rates.

**Results:**

Of 1840 patients, 1693 (92.0%) sustained isolated pelvic fractures and 147 (8.0%) had concomitant UE fractures. Groups were comparable at baseline across all measured covariates. LOS did not differ significantly between groups (11.0 ± 14.2 vs. 11.8 ± 16.9 days; *p* = 0.796). Mortality was similar at all evaluated time points, including 30-day (3.3% vs. 3.6%; *p* = 0.752) and 1-year mortality (10.9% vs. 14.3%; *p* = 0.452). Readmission rates and systemic infection rates were comparable. Subgroup analyses by UE fracture location, treatment approach, and pelvic fracture type demonstrated no significant differences.

**Conclusions:**

Concomitant upper extremity fractures do not adversely affect clinical outcomes in geriatric patients with conservatively managed pelvic fractures. The contrast between these findings and published data in surgically managed proximal femur fracture patients supports the hypothesis that surgical complexity, rather than rehabilitative impairment alone, is the primary driver of adverse outcomes in elderly patients with concurrent fractures.

## Introduction

Pelvic fractures in elderly patients represent a growing and increasingly recognized source of morbidity in the aging population [1].Predominantly caused by low-energy falls in the context of osteoporotic bone, fragility fractures of the pelvic ring, involving the pubic rami, sacrum, and ilium, along with acetabular fractures, are associated with severe pain, impaired mobility, prolonged hospitalization, and functional decline [2], [3]. Reported one-year mortality rates following conservatively managed pelvic ring fractures range from 10% to 27%, reflecting both the severity of the injury and the frailty of the affected population [4]. Unlike femoral neck and intertrochanteric fractures, which mandate operative intervention in the majority of eligible patients, pelvic ring fractures in the elderly are predominantly managed conservatively through analgesia, early mobilization, and physical rehabilitation [1], [5]. Acetabular fractures, while more variable in their management, are similarly treated conservatively in a substantial proportion of elderly patients due to the high operative risk and technical complexity of surgical fixation in this population [6]. In this conservative management paradigm, intact upper extremity function is indispensable, patients rely heavily on walking frames, crutches, and other assistive devices to facilitate weight-bearing and early ambulation during recovery, and upper limb strength is a key determinant of post-fracture rehabilitation success [7].

A clinically important subset of geriatric fracture patients sustains concurrent upper extremity (UE) fractures at the time of their index injury, estimated to occur in 5–8% of cases in the proximal femur fracture population [8], [9], [10]. Theoretically, the presence of a concomitant UE fracture is of particular concern in patients whose recovery depends on upper limb weight-bearing capacity. Impaired grip strength, restricted elbow or shoulder range of motion, or the necessity for upper limb immobilization may limit effective use of assistive devices, potentially delaying ambulation and rehabilitation, prolonging hospitalization, and increasing susceptibility to immobilization-associated complications such as venous thromboembolism, pneumonia, and deconditioning [11]. These concerns have biological plausibility, and in the context of surgically managed proximal femur fractures, prior studies have demonstrated associations between concomitant UE fractures and prolonged hospitalization, increased morbidity, and in some series, higher mortality rates [8], [12], [13].

Existing literature on concomitant upper extremity fractures in geriatric patients has focused almost exclusively on operatively managed proximal femur fractures [13], [14]. However, this setting differs substantially from conservatively managed pelvic fractures, where surgical factors are absent and the potential impact of a concomitant upper extremity fracture on outcomes remains unclear.

The primary objective of this study was to determine the independent impact of concomitant upper extremity fractures on hospital length of stay and all-cause mortality in geriatric patients with conservatively managed pelvic and acetabular fractures. Subgroup analyses examined the effect of UE fracture location and treatment approach. Secondary outcomes included hospital re-admission and systemic infection rates at predefined time points. We hypothesized that concomitant UE fractures would be associated with longer hospitalization and higher complication rates compared with isolated pelvic fractures.

## Methods

### Study design and population

A retrospective cohort study was conducted using electronic medical records from a Level 1 trauma center. Following Institutional Review Board approval, all patients aged ≥ 65 years admitted with a primary diagnosis of pelvic ring or acetabular fracture between January 2010 and January 2024 were identified. This study was conducted and reported in accordance with the Strengthening the Reporting of Observational Studies in Epidemiology (STROBE) guidelines [15].

Inclusion criteria were: (1) age ≥ 65 years at the time of fracture; (2) primary diagnosis of pelvic ring or acetabular fracture; (3) non-operative management of the pelvic or acetabular injury; (4) complete demographic data; and (5) minimum 12-month follow-up or confirmed death within the study period. Exclusion criteria included: (1) pathological fractures secondary to malignancy; (2) periprosthetic fractures; (3) patients transferred from other institutions with incomplete initial assessment data; and (4) pre-existing non-ambulatory status prior to the injury.

The main exposure variable was the presence of a concomitant UE fracture, defined as any radiographically confirmed fracture of the humerus, radius, ulna, or carpal bones sustained during the same traumatic event as the pelvic fracture. Patients were classified into two groups: those with isolated pelvic or acetabular fractures (isolated group) and those with concomitant UE fractures (concomitant group). It is important to note that “non-operative management” refers specifically to the treatment of the pelvic or acetabular injury; some patients in the concomitant group underwent operative treatment for their UE fracture, which is described in the relevant subgroup analysis.

Primary outcomes were hospital length of stay (LOS), defined as total days from admission to discharge for the index hospitalization, and all-cause mortality assessed at in-hospital, 7-day, 30-day, 90-day, and 1-year time points from the date of fracture diagnosis. Mortality was derived from electronic vital status records; for each patient, the duration from fracture date to death (for deceased patients) or to last follow-up (for surviving patients) was recorded and used as the primary reference for time-point mortality derivation. Secondary outcomes comprised all-cause hospital readmission at 7, 30, and 90 days post-discharge, and the occurrence of systemic infections, including urinary tract infections (UTI), bacteremia, and pneumonia, documented during the follow-up period.

Covariates recorded at the time of admission included: age, sex, body mass index (BMI), Charlson Comorbidity Index (CCI) score, ASA physical status classification, pelvic fracture type (pelvic ring vs. acetabular), and specific comorbidities including chronic obstructive pulmonary disease (COPD), congestive heart failure (CHF), diabetes mellitus, chronic kidney disease, and prior myocardial infarction.

### Statistical analysis

Descriptive statistics were calculated for all variables. Continuous variables are presented as mean ± standard deviation (SD) or median [interquartile range (IQR)], depending on the distribution of the data as assessed by the Shapiro-Wilk test and visual inspection of histograms. Between-group comparisons used the Mann-Whitney U test for continuous variables and the chi-squared or Fisher’s exact test for categorical variables, as appropriate. Subgroup analyses were performed by UE fracture location (distal radius, proximal humerus, olecranon, and other), UE fracture treatment approach (operative vs. non-operative), and pelvic fracture type (pelvic ring vs. acetabular). For comparisons of LOS across multiple subgroups, the Kruskal-Wallis test was applied. Statistical significance was set at a two-tailed p-value < 0.05. All analyses were performed using SPSS Statistics software version 29 (IBM SPSS, New York, USA).

## Results

### Study cohort and baseline characteristics

A total of 1,840 patients met the inclusion criteria and were analyzed: 1,693 (92.0%) with isolated pelvic or acetabular fractures and 147 (8.0%) with concomitant UE fractures. Among patients in the concomitant group, the most common UE fracture locations were proximal humerus (*n* = 61, 41.5%), distal radius (*n* = 43, 29.3%), and olecranon (*n* = 27, 18.4%), with humerus shaft (*n* = 9, 6.1%) and carpal bones (*n* = 7, 4.8%) occurring less frequently. Pelvic ring fractures predominated in both groups (82.1% and 83.0%, respectively), with the remainder comprising acetabular fractures. Of the 147 patients with concomitant UE fractures, 131 (89.1%) were managed non-operatively for their UE injury, while 16 (10.9%) underwent operative fixation, comprising olecranon ORIF (*n* = 9), distal radius ORIF (*n* = 4), humeral shaft ORIF (*n* = 2), and shoulder arthroplasty (*n* = 1).

Baseline characteristics were well matched between groups (Table 1). Mean age was 82.2 ± 7.9 years in the isolated group and 81.0 ± 8.4 years in the concomitant group (*p* = 0.068). Female sex predominated in both groups (76.0% vs. 80.3%, *p* = 0.281). CCI scores were comparable (3.9 ± 1.5 vs. 3.9 ± 1.3, *p* = 0.827), and no significant differences were observed in the distribution of individual comorbidities. BMI and ASA scores were available in fewer than 26% of patients and were therefore reported descriptively only; they were excluded from between-group comparisons due to the extent of missingness. Follow-up duration was marginally longer in the concomitant group (27.3 ± 34.8 vs. 20.4 ± 30.5 months, *p* = 0.035); since this would provide greater opportunity to detect events in the concomitant group, it does not bias toward a null finding. All analyses were performed using all patients for whom the relevant outcome data were available. LOS was available for 1,225 patients (66.5%); vital status and time to death or last follow-up were available for 1,062 patients (57.7%). Missingness of LOS was non-differential between groups (33.3% vs. 34.7%, *p* = 0.803), and patients with and without LOS data were demographically comparable. Mortality data were unavailable for a substantial proportion of patients admitted in more recent years, reflecting insufficient follow-up duration rather than data loss, a pattern confirmed by the strong temporal correlation between year of admission and completeness of vital status data (Spearman *r* = 0.96); accordingly, a complete-case restriction would have introduced systematic bias by selectively excluding recently admitted, younger patients who had not yet had sufficient time to accumulate mortality events.

**Table 1:** Baseline characteristics of geriatric patients with pelvic fractures stratified by presence of concomitant upper extremity fracture

Characteristic	Isolated pelvic fracture (*n* = 1693)	Concomitant UE Fracture (*n* = 147)	*p*-value
Demographics			
Age (years), mean ± SD	82.2 ± 7.9	81.0 ± 8.4	0.068
Female sex, n (%)	1286 (76.0%)	118 (80.3%)	0.281
Follow-up duration (months), mean ± SD	20.4 ± 30.5	27.3 ± 34.8	0.035
Fracture characteristics			
Pelvic ring fracture, n (%)	1390 (82.1%)	122 (83.0%)	0.874
Acetabular fracture, n (%)	303 (17.9%)	25 (17.0%)	–
Clinical characteristics			
BMI (kg/m²), mean ± SD	22.0 ± 8.5 (*n* = 418)	22.1 ± 9.2 (*n* = 50)	0.518
Charlson comorbidity index, mean ± SD	3.9 ± 1.5 (*n* = 1,487)	3.9 ± 1.3 (*n* = 127)	0.827
CCI 1–2 (mild), n (%)	197/1487 (13.2%)	13/127 (10.2%)	0.406
CCI 3–4 (moderate), n (%)	889/1487 (59.8%)	78/127 (61.4%)	0.790
CCI ≥ 5 (severe), n (%)	401/1487 (27.0%)	36/127 (28.3%)	0.817
ASA score, mean ± SD	2.7 ± 0.7 (*n* = 415)	2.7 ± 0.7 (*n* = 44)	0.785
ASA score ≥ 3, n (%)	250/415 (60.2%)	27/44 (61.4%)	1.000
Comorbidities			
COPD, n (%)	55/1487 (3.7%)	6/127 (4.7%)	0.473
Congestive heart failure, n (%)	41/1487 (2.8%)	3/127 (2.4%)	1.000
Diabetes mellitus, n (%)	197/1487 (13.2%)	17/127 (13.4%)	1.000
Chronic kidney disease, n (%)	54/1487 (3.6%)	1/127 (0.8%)	0.121
Prior myocardial infarction, n (%)	12/1485 (0.8%)	2/127 (1.6%)	0.303

*SD *Standard aeviation,* BMI *Body mass index,* CCI *Charlson Comorbidity Index, *ASA* American society of anesthesiologists,* COPD *Chronic obstructive pulmonary disease,* UE *Upper extremity

### Primary outcomes

LOS did not differ significantly between groups. Mean LOS was 11.0 ± 14.2 days (median 5.9 [IQR 2.8–12.7] days; *n* = 1,129) in the isolated group and 11.8 ± 16.9 days (median 5.2 [IQR 2.6–12.4] days; *n* = 96) in the concomitant group (*p* = 0.796).

Mortality rates were comparable between groups at all evaluated time points. In-hospital mortality was 1.0% (7 of 728 assessable patients) in the isolated group and 1.6% (1 of 61) in the concomitant group (*p* = 0.476). Seven-day mortality was 1.5% vs. 2.4% (*p* = 0.638), 30-day mortality was 3.3% vs. 3.6% (*p* = 0.752), and 90-day mortality was 6.1% vs. 7.1% (*p* = 0.895). One-year mortality was 10.9% (107 of 978) in the isolated group and 14.3% (12 of 84) in the concomitant group (*p* = 0.452) Table 2.

**Table 2:** Primary and secondary outcomes

Outcome	Isolated pelvic fracture (*n* = 1693)	Concomitant UE fracture (*n* = 147)		*p*-value
Primary outcomes				
Length of stay (days) Mean ± SD Median [IQR]	11.0 ± 14.2 5.9 [2.8–12.7] (*n* = 1,129)		11.8 ± 16.9 5.2 [2.6–12.4] (*n* = 96)	0.796
Mortality, n (%)				
In-hospital	7/728 (1.0%)		1/61 (1.6%)	0.476
7-day	15/978 (1.5%)		2/84 (2.4%)	0.638
30-day	32/978 (3.3%)		3/84 (3.6%)	0.752
90-day	60/978 (6.1%)		6/84 (7.1%)	0.895
1-year	107/978 (10.9%)		12/84 (14.3%)	0.452
Secondary outcomes				
Readmission, n (%)				
7-day	61/1693 (3.6%)		6/147 (4.1%)	0.946
30-day	159/1693 (9.4%)		14/147 (9.5%)	1.000
90-day	283/1693 (16.7%)		20/147 (13.6%)	0.390
Systemic Infections, n (%)				
Any systemic infection	240/1693 (14.2%)		17/147 (11.6%)	0.452
Urinary tract infection	165/1693 (9.7%)		14/147 (9.5%)	1.000
Bacteremia	62/1693 (3.7%)		2/147 (1.4%)	0.220
Pneumonia	13/1693 (0.8%)		1/147 (0.7%)	1.000

*UE *Upper extremity,* SD *Standard deviation,* IQR *Interquartile range

### Secondary outcomes

Readmission rates were similar between groups at all time points (Table 2). Seven-day readmission occurred in 3.6% and 4.1% of isolated and concomitant patients, respectively (*p* = 0.946). Thirty-day readmission rates were 9.4% and 9.5% (*p* = 1.00), and 90-day readmission rates were 16.7% and 13.6% (*p* = 0.390).

Systemic infection rates did not differ significantly between groups. Any systemic infection was recorded in 14.2% of isolated patients and 11.6% of concomitant patients (*p* = 0.452). Urinary tract infection was the most common infection type in both groups (9.7% vs. 9.5%, *p* = 1.00). Bacteremia occurred in 3.7% of the isolated group and 1.4% of the concomitant group (*p* = 0.220), and pneumonia rates were 0.8% vs. 0.7% (*p* = 1.00), respectively.

### Subgroup analyses

Subgroup analysis by UE fracture location demonstrated no significant differences in any outcome when comparing individual fracture locations to the isolated group (Table 3). LOS was similar across all location subgroups. A nominally significant difference in LOS was observed in the humerus shaft subgroup (mean 19.8 ± 9.1 vs. 11.0 ± 14.2 days, *p* = 0.048); however, this comparison is based on only four patients with available LOS data and cannot be considered a meaningful finding. Thirty-day mortality, 1-year mortality, 90-day readmission, and infection rates showed no significant differences across any UE fracture location.

**Table 3:** Outcomes by location of concomitant upper extremity fracture

Outcome	Distal radius (*n* = 43)	Proximal humerus (*n* = 61)	Olecranon (*n* = 27)	Other UE # (*n* = 16)	Isolated pelvic (*n* = 1693)
LOS (days) mean ± SD	9.4 ± 11.8	9.1 ± 14.0	19.0 ± 26.3	16.8 ± 17.8	11.0 ± 14.2
p-value vs. isolated	0.532	0.228	0.160	0.068 ‡	–
30-day mortality, n (%)	0/22 (0.0%)	2/32 (6.2%)	1/19 (5.3%)	0/8 (0.0%)	32/978 (3.3%)
p-value vs. isolated	1.000	0.293	0.476	1.000	–
1-year mortality, n (%)	2/22 (9.1%)	6/32 (18.8%)	2/19 (10.5%)	2/8 (25.0%)	107/978 (10.9%)
p-value vs. isolated	1.000	0.159	1.000	0.218	–
90-day readmission, n (%)	7/43 (16.3%)	7/61 (11.5%)	5/27 (18.5%)	1/16 (6.3%)	283/1,693 (16.7%)
p-value vs. isolated	1.000	0.364	0.795	0.464	–
Any infection, n (%)	5/43 (11.6%)	5/61 (8.2%)	5/27 (18.5%)	2/16 (12.5%)	240/1,693 (14.2%)
p-value vs. isolated	0.801	0.256	0.575	1.000	–

p-values compare each concomitant location subgroup against the isolated pelvic fracture group

‡ *p* = 0.048 for humerus shaft LOS comparison, based on *n* = 4 patients with available LOS data. # Other UE locations: humerus shaft (*n* = 9) and carpal bones (*n* = 7). *UE* upper extremity

When outcomes were stratified by UE fracture treatment approach, patients who underwent operative fixation of their UE injury (*n* = 16) had a longer mean LOS compared to both the isolated group (16.8 ± 27.1 vs. 11.0 ± 14.2 days, *p* = 0.530) and the non-operative UE management group (16.8 ± 27.1 vs. 11.0 ± 15.0 days, *p* = 0.369). Neither comparison reached statistical significance. The operative UE subgroup comprises only 16 patients; these results are presented as descriptive observations only and no inferential conclusions can be drawn.

Stratified analysis by pelvic fracture type revealed consistent null findings in both the pelvic ring and acetabular subgroups (Table 4). In neither subgroup did the presence of a concomitant UE fracture significantly affect LOS, 30-day mortality, 1-year mortality, 90-day re-admission, or systemic infection rates.

**Table 4:** Outcomes stratified by pelvic fracture type

Outcome	Pelvic ring isolated (*n* = 1,390)	Pelvic ring concomitant (*n* = 122)	*p*	Acetabular isolated (*n* = 303)	Acetabular concomitant (*n* = 25)	*p*
LOS (days), mean ± SD	10.4 ± 12.4	12.3 ± 17.7	0.938	14.2 ± 20.2	7.2 ± 5.9	0.440
30-day mortality, n (%)	23/814 (2.8%)	2/69 (2.9%)	1.000	9/164 (5.5%)	1/15 (6.7%)	0.593
1-year mortality, n (%)	83/814 (10.2%)	9/69 (13.0%)	0.416	24/164 (14.6%)	3/15 (20.0%)	0.704
90-day readmission, n (%)	244/1,390 (17.6%)	17/122 (13.9%)	0.374	39/303 (12.9%)	3/25 (12.0%)	1.000
Any infection, n (%)	187/1,390 (13.5%)	16/122 (13.1%)	1.000	53/303 (17.5%)	1/25 (4.0%)	0.095

*UE *Upper extremity,* SD *Standard deviation

## Discussion

This retrospective cohort study of 1,840 geriatric patients with conservatively managed pelvic and acetabular fractures demonstrates that concomitant upper extremity fractures are not independently associated with longer hospital stays, increased mortality at any time point, higher readmission rates, or greater rates of systemic infection. These findings were consistent across all subgroup analyses by UE fracture location, UE fracture treatment approach, and pelvic fracture type, and were observed in groups that were well matched for age, comorbidity burden, sex, and fracture characteristics. The published literature on concomitant UE fractures in geriatric fracture patients derives exclusively from studies of operatively managed proximal femur fractures. Sharrock et al. [8] reported in a systematic review and meta-analysis of 210,289 patients that concurrent UE fractures were associated with significantly longer hospital stays following operative proximal femur fracture management. Kang et al. [10] and Thayer et al. [12] similarly demonstrated associations between concomitant UE fractures and adverse outcomes in this surgically managed population. More recently, Graif et al. [13] reported in a contemporary cohort of 7,488 operatively treated proximal femur fracture patients that concomitant UE fractures independently predicted a 2.7-day increase in LOS, with operative management of the UE fracture associated with substantially longer hospitalizations and a markedly higher risk of revision surgery. Lee et al. [14] further demonstrated that the surgical management of concomitant UE fractures significantly impacted outcomes in this context. The present study, using a comparable design, population, and institution, finds no evidence of equivalent harm in the conservative pelvic fracture setting.

This divergence from the proximal femur fracture literature has a plausible explanation. In the operative setting, concomitant UE fractures introduce a series of surgical complexities that are entirely absent in conservative pelvic fracture management: dual anesthetic exposure, compromised intraoperative positioning for proximal femur fixation or arthroplasty, sequential operative procedures often performed within days of each other, and compounded physiological stress in an already frail and medically complex patient [12]. The present findings suggest that once these surgical elements are removed, the rehabilitative burden of a UE fracture, impaired walker use, limited grip strength, restricted upper limb mobility, is insufficient in isolation to measurably alter hospitalization duration or clinical outcomes in patients with conservatively managed pelvic fractures. This is consistent with observations by Di Monaco et al. [16], who found that the anatomical site of UE injury may be less influential than originally hypothesized, and with the work of Lübbeke et al. [11], who demonstrated that UE fractures in elderly patients increase rehabilitation resource utilization without necessarily translating to measurably worse mortality outcomes. Importantly, 89.1% of concomitant UE fractures in our cohort were managed non-operatively, implying relatively stable injuries that may have permitted earlier adaptive rehabilitation strategies and modified assistive device use.

The subgroup analysis by UE treatment approach is noteworthy despite its substantial limitations. The 16 patients who underwent operative fixation of their UE fracture demonstrated a numerically longer LOS (16.8 vs. 11.0 days) that is directionally consistent with the surgical complexity hypothesis. This observation, though statistically non-significant given the small sample size, aligns with findings from the proximal femur fracture literature and provides a hypothesis-generating signal: even in the context of conservative pelvic fracture management, the addition of operative UE fixation may introduce sufficient surgical complexity to influence the clinical trajectory [13]. A dedicated, adequately powered study is required to examine this subgroup rigorously.

These findings carry practical implications for the clinical management of elderly patients presented with pelvic fractures. The presence of a concomitant UE fracture should not independently drive escalations in resource allocation, intensive care admission thresholds, or clinical risk stratification in this population. The primary determinants of clinical outcome remain the nature and severity of the pelvic injury, the patient’s premorbid functional status and comorbidity burden, and the quality of conservative management and orthogeriatric care, consistent with established evidence on fragility pelvic ring fractures [1], [5]. This conclusion should not be misinterpreted as diminishing the clinical importance of the UE fracture, which warrants appropriate management, pain control, and functional rehabilitation. Rather, it suggests that in the conservative pelvic fracture context, the presence of a concomitant UE injury does not independently compound the clinical course in a measurable way.

This study has several limitations that should be considered when interpreting the results. As a retrospective single-center study, it is subject to inherent selection bias and the findings may not be fully generalizable to institutions with different patient demographics, treatment protocols, or orthogeriatric care models. LOS data was available for 66.5% of patients; while missingness was non-differential between groups and patients with and without LOS data were demographically comparable, the substantial proportion of unavailable records reflects gaps in early administrative data from the first years of the study period. Vital status and time-to-event data were unavailable for 42.3% of patients, predominantly those admitted in more recent years with insufficient follow-up duration to ascertain mortality; reported mortality rates should therefore be interpreted in the context of a partially censored dataset. The operative UE fracture subgroup comprised only 16 patients, rendering any statistical analysis of this clinically important comparison severely underpowered; the descriptive findings presented are hypothesis-generating only. Functional outcome data, including ambulatory status at discharge, discharge destination, quality of life measures, and validated rehabilitation scores were not available in the electronic record; these measures are essential to characterize the full rehabilitative impact of concomitant UE fractures and represent a critical limitation of the present analysis. BMI and ASA physical status score were available in fewer than 26% of patients, precluding full multivariable adjustment for frailty and physiological reserve, both of which are recognized predictors of outcome in elderly fracture patients. Finally, readmission and infection data represent event-recording, and patients who sought care at other institutions would not have been captured in the institutional record, potentially resulting in underestimation of these outcomes.

## Conclusions

Concomitant upper extremity fractures do not adversely affect hospital length of stay, all-cause mortality, readmission rates, or systemic infection rates in geriatric patients with conservatively managed pelvic and acetabular fractures. The absence of this effect contrasts with published findings in operatively managed proximal femur fracture patients, supporting the hypothesis that surgical complexity, rather than rehabilitative impairment alone, is the primary driver of adverse outcomes in elderly patients with concurrent fractures.

## Data Availability

Data are available at reasonable request from the corresponding author.

## References

[1] Küper MA, Trulson A, Stuby FM, Stöckle U (2019) Pelvic ring fractures in the elderly. EFORT Open Rev 4(6):313–320. 10.1302/2058-5241.4.180062 31312519 10.1302/2058-5241.4.180062 PMC6598730

[2] Ghassibi M, Boyalakuntla DS, Gentile J (2019) Low-energy pelvic ring fractures in the elderly population: expected outcomes and associated mortality rates. J Clin Med Res 11(11):725–728. 10.14740/jocmr3891 31803314 10.14740/jocmr3891 PMC6879025

[3] Schmitz P, Lüdeck S, Baumann F, Kretschmer R, Nerlich M, Kerschbaum M (2019) Patient-related quality of life after pelvic ring fractures in elderly. Int Orthop 43(2):261–267. 10.1007/s00264-018-4030-8 29946740 10.1007/s00264-018-4030-8

[4] Timmer RA, Verhage SM, Krijnen P, Meylaerts SAG, Schipper IB (2023) Indications for surgical fixation of low-energy pelvic ring fractures in elderly: a systematic review. Arch Orthop Trauma Surg 143(5):2417–2428. 10.1007/s00402-022-04438-w 35462589 10.1007/s00402-022-04438-w PMC10110636

[5] Papakostidis KCG, Giannoudis PV (2025) Evolution of treatment of fragility fractures of the pelvic ring. An update. Injury 56(2):112145. 10.1016/j.injury.2025.112145 39847824 10.1016/j.injury.2025.112145

[6] Vipulendran K, Kelly J, Rickman M, Chesser T (2021) Current concepts: managing acetabular fractures in the elderly population. Eur J Orthop Surg Traumatol 31(5):807–816. 10.1007/s00590-021-02931-3 33772661 10.1007/s00590-021-02931-3

[7] Rommens PM, Hofmann A (2023) The FFP-classification: from eminence to evidence. Injury 54(Suppl 3):S10–S19. 10.1016/j.injury.2021.09.016 34598791 10.1016/j.injury.2021.09.016

[8] Sharrock M, Hagan J, Lee J, Charalambous CP (2022) Greater length of hospital stay for concurrent hip and upper limb fractures compared to isolated hip fractures: a systematic review of 13 studies including 210,289 patients and meta-analysis. Injury 53(7):2617–2624. 10.1016/j.injury.2022.05.039 35644643 10.1016/j.injury.2022.05.039

[9] Mulhall KJ, Ahmed A, Khan Y, Masterson E (2002) Simultaneous hip and upper limb fracture in the elderly: incidence, features and management considerations. Injury 33(1):29–31. 10.1016/s0020-1383(01)00097-3 11879829 10.1016/s0020-1383(01)00097-3

[10] Kang SW, Shin WC, Moon NH, Suh KT (2019) Concomitant hip and upper extremity fracture in elderly patients: prevalence and clinical implications. Injury 50(11):2045–2048. 10.1016/j.injury.2019.09.010 31543316 10.1016/j.injury.2019.09.010

[11] Lübbeke A, Stern R, Grab B, Herrmann F, Michel JP, Hoffmeyer P (2005) Upper extremity fractures in the elderly: consequences on utilization of rehabilitation care. Aging Clin Exp Res 17(4):276–80. 10.1007/BF03324610 16285192 10.1007/BF03324610

[12] Thayer MK, Kleweno CP, Lyons VH, Taitsman LA (2018) Concomitant upper extremity fracture worsens outcomes in elderly patients with hip fracture. Geriatr Orthop Surg Rehabil 9:2151459318776101. 10.1177/2151459318776101 29900029 10.1177/2151459318776101 PMC5992804

[13] Graif N, Elbaz E, Warschawski Y, Kazum E, Shabtai L, Amzallag N, Factor S (2025) The impact of concomitant upper extremity fractures on outcomes in geriatric patients following hip fracture surgery. J Clin Med 14(18):6380. 10.3390/jcm14186380 41010586 10.3390/jcm14186380 PMC12470443

[14] Lee MJ, Ng J, Kok TWK, Kwek BKE (2023) Does the surgical treatment of concomitant upper limb fractures affect the outcomes of hip fractures in the elderly population? Arch Orthop Trauma Surg 143(1):353–358. 10.1007/s00402-021-04328-7 34985565 10.1007/s00402-021-04328-7

[15] Von Elm E, Altman DG, Egger M, Pocock SJ, Gøtzsche PC, Vandenbroucke JP (2007) The strengthening the reporting of observational studies in epidemiology (STROBE) statement: guidelines for reporting observational studies. STROBE initiative. Lancet. 10.1016/S0140-6736(07)61602-X 10.1136/bmj.39335.541782.AD PMC2034723 17947786

[16] Di Monaco M, Castiglioni C, Vallero F, Di Monaco R, Tappero R (2015) Concomitant upper limb fractures and short-term functional recovery in hip fracture patients: does the site of upper limb injury matter? Am J Phys Med Rehabil. 10.1097/PHM.0000000000000191 25251255 10.1097/PHM.0000000000000191

